# Monitoring transcription initiation activities in rat and dog

**DOI:** 10.1038/sdata.2017.173

**Published:** 2017-11-28

**Authors:** Marina Lizio, Abdul Kadir Mukarram, Mizuho Ohno, Shoko Watanabe, Masayoshi Itoh, Akira Hasegawa, Timo Lassmann, Jessica Severin, Jayson Harshbarger, Imad Abugessaisa, Takeya Kasukawa, Chung Chau Hon, Piero Carninci, Yoshihide Hayashizaki, Alistair R.R. Forrest, Hideya Kawaji

**Affiliations:** 1 RIKEN Center for Life Science Technologies, Division of Genomic Technologies, Yokohama 230-0045, Japan; 2 RIKEN Yokohama Institute, Omics Science Center, Yokohama 230-0045, Japan; 3 Department of Biosciences and Nutrition, Karolinska Institutet, Stockholm SE-171 76, Sweden; 4 RIKEN Preventive Medicine and Diagnosis Innovation Program, Wako 351-0198, Japan; 5 Telethon Kids Institute, Telethon Kids Institute, The University of Western Australia, Subiaco WA 6008, Australia; 6 Harry Perkins Institute of Medical Research, Nedlands WA 6009, Australia; 7 RIKEN Advanced Center for Computing and Communication, Preventive Medicine and Applied Genomics Unit, Yokohama 230-0045, Japan

**Keywords:** Data processing, Research data, Transcriptomics, Computational platforms and environments

## Abstract

The promoter landscape of several non-human model organisms is far from complete. As a part of FANTOM5 data collection, we generated 13 profiles of transcription initiation activities in dog and rat aortic smooth muscle cells, mesenchymal stem cells and hepatocytes by employing CAGE (Cap Analysis of Gene Expression) technology combined with single molecule sequencing. Our analyses show that the CAGE profiles recapitulate known transcription start sites (TSSs) consistently, in addition to uncover novel TSSs. Our dataset can be thus used with high confidence to support gene annotation in dog and rat species. We identified 28,497 and 23,147 CAGE peaks, or promoter regions, for rat and dog respectively, and associated them to known genes. This approach could be seen as a standard method for improvement of existing gene models, as well as discovery of novel genes. Given that the FANTOM5 data collection includes dog and rat matched cell types in human and mouse as well, this data would also be useful for cross-species studies.

## Background & Summary

The recent years have seen a renewed interest in non-human model organisms, mainly thanks to the advances in DNA sequencing technologies. Even though next generation sequencing made the most disparate genomes available^1,2^, building correct gene models either by de-novo or alignment-based assembly, remains difficult. That is mainly due to the inability to accurately map very long transcripts (i.e., >10 kb)^3–6^.

Defining gene models, though, is one of the main steps in the characterization of an organism’s DNA repertoire. Comparison of genome sequences across multiple organisms is one powerful approach to uncover evolutionary gain/loss and constraints of genetically inherited information, thus shedding a light not only on evolution but also on disease^7^. Animal model organisms, as a matter of fact, have been broadly used in research under the assumption that understanding the systems in these organisms would translate into understanding the same systems in human. This is of particular importance when human experimentation is unfeasible or unethical. However, all genomic studies rely on accurate gene models in order to locate functional elements in genomes and assess the impact of conservation. The lack of correct and comprehensive genome annotations impedes further progresses in the field.

Among the technologies that allow identification of transcripts, RNA-seq and Cap Analysis of Gene Expression (CAGE)^8^ paired with sequencing are the most widely used^9,10^. They both reveal the presence and the amount of RNAs in a given biological sample at a given state, but their final readout is different. RNA-seq facilitates the determination of intron/exon boundaries and alternative splicing, even though with some limitations in defining precise 5′ and 3′ termini. CAGE, although it cannot generate the entire structure of RNAs, can explain promoter usage and architecture due to its ability to map capped 5′-ends of transcripts with high accuracy. Moreover, CAGE can detect promoters of capped ncRNAs, making it a useful tool to study antisense transcription, or to identify actively transcribed enhancer RNAs^11^, thus providing immediate insights on gene regulation. As shown by recent findings on human long noncoding RNAs (lncRNAs)^12^, complementing CAGE- and RNA-seq approaches can contribute significantly to improve the definition of gene models.

The functional annotation of the mammalian genome (FANTOM) project aimed at identifying functional elements of genomes^9,13–15^. Within FANTOM5, the fifth collaborative effort of the fifteen-year-long project, CAGE was applied to nearly 2,000 human and 1,000 mouse primary cell, tissue and cellular states of differentiation and stimulation types, to generate promoter atlases of unprecedented coverage, by using the same technology and the same platform^16,17^. The outcomes of this large-scale effort include: gene expression quantification across all samples, promoters at a high resolution, bi-directionally transcribed enhancers, transcription factor activities, and co-expression networks of genes. All the data, raw and processed, were further integrated into a public web resource^18^ for the benefit of the scientific community (http://fantom.gsc.riken.jp/). Partner web browsers and other web tools are also accessible from our gateway site^19–24^.

**Figure 1:**
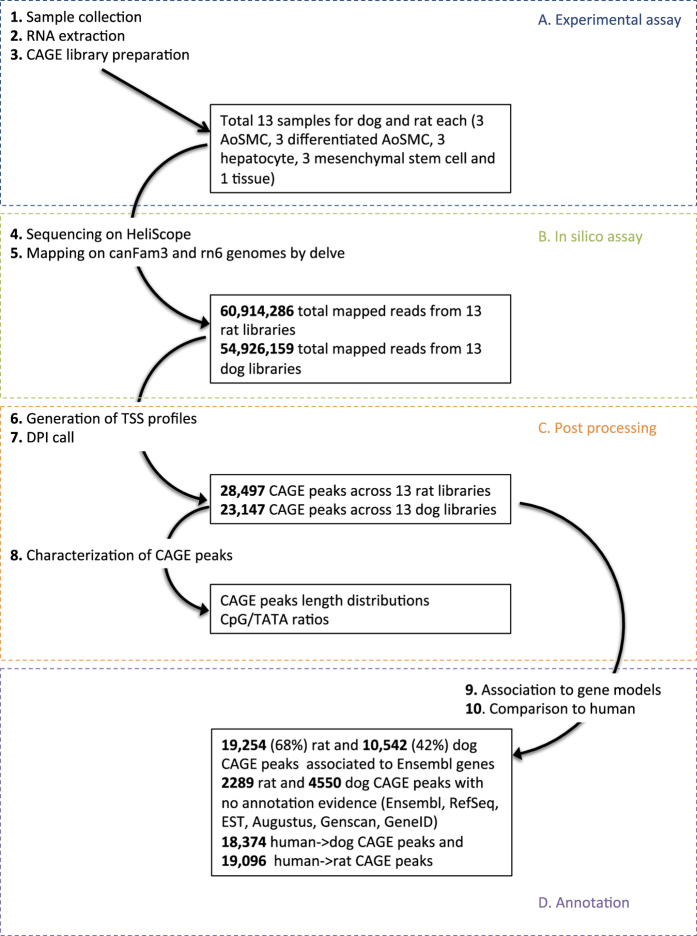
Study overview. The steps of sample collection, CAGE data production, post-processing and further analyses are shown as arrows with their results indicated by squares.

The human (*Homo sapiens*) and mouse (*Mus musculus*) genomes have been extensively studied and have well-characterized transcriptomes^13,14,16,25^. Other mammalian genomes, however, have not benefited from this level of in-depth analyses. Therefore, in order to improve the definition of gene models for the less-studied organisms, the FANTOM consortium generated CAGE data for several other species, like dog (*Canis lupus familiaris*) and rat (*Rattus norvegicus*). Experimentally defined transcriptome in these species will provide a baseline for cross-species studies of functional genomics and medical researches using disease models. From each species, we profiled 13 samples representative of three primary cell types matching human, mouse and chicken (*Gallus gallus*) primary cells. We identified CAGE peaks and associated them to the existing gene models as a part of our quality assessment; this also highlighted the utility of CAGE to refine incorrectly characterized gene models. Although the number of collected samples is meagre and not representative of all cell types, the same cell types are profiled with the same technology across 5 species, providing opportunities to understand similar cellular systems and functions across multiple organisms, and to study cell-type-specific promoters architecture and evolution. The full sets of CAGE promoters are available both via DDBJ data repository (mapping results) and via the FANTOM web resource (expression and genomic visualization), which can be accessible at http://fantom.gsc.riken.jp. A schematic of the entire workflow is summarised in Fig. 1.

## Methods

### Sample collection and RNA extraction

Samples used in this study were collected in order to allow comparison of promoter sets and transcriptional regulatory models across several organisms. They were chosen in order to represent varied cells, such as derived from different germ layers, and cells with self-renewal potential. For both beagle dog (*Canis lupus familiaris*) and rat (*Rattus norvegicus*), a total of 13 matched samples each were used. One universal RNA tissue sample and 12 RNA primary cell samples from three biological replicates (6 aortic smooth muscle cell, 3 hepatocyte and 3 mesenchymal stem cell) were purchased. Specifically, universal RNA tissue for both dog and rat was obtained from Biochain (Newark, CA, USA); aortic smooth muscle and mesenchymal cells of dog and of all 12 rat primary cell samples from Cell Applications (San Diego, CA, USA); dog hepatocytes from Celsis (Chicago, IL, USA) and BD Gentest (Franklin Lakes, NJ, USA). The rat samples purchased were derived from Sprague Dawley strain, except two aortic smooth muscle cell samples that were derived from Lewis strain rats. We obtained two sets of aortic smooth muscle cells for both dog and rat, depending on which medium they are kept in; one set is kept to proliferate in a myoblast-like state, while the other set is made differentiate towards myotubes (where the suffix ‘diff’ is used to tell them apart). Total RNA was extracted using miRNeasy kit (QUIAGEN Valencia, CA, USA), following manufacturer’s instructions. Age or developmental stage information for neither dog nor rat samples could be obtained. A detailed list of all samples used in this study is available in Supplementary Table 1.

### Library preparation and sequencing

CAGE libraries were prepared for single molecule sequencing as described previously^26^. Two protocols were applied for the preparation of dog samples. The standard library preparation method used 5 ug of total RNA, whereas a low quantity protocol was used for a sample of 1 ug or less total RNA (dog aortic smooth muscle cells donor1). CAGE libraries were subsequently sequenced on HeliScope sequencers^27^, following the manufacturer’s instructions.

### Mapping and data processing

Sequenced libraries were first filtered for reads aligning to ribosomal DNA with up to two mismatches and for artifacts. We used our own developed tool Tag-dust^28^ to remove artifactual sequences. Retained reads were aligned to canFam3 (Sept 2011, Broad canFam3.1/canFam3) and rn6 (July 2014, RGSC 6.0/rn6) reference genomes by delve (see Code availability), which leverages on a hidden markov model (HMM) to assign probability scores to all mapped positions in the genome. It then uses all the probability scores obtained to calculate the most likely alignment. Mapping of each library was achieved by performing the following commands:

*‘delve seed -l 12 -s 8 -o SEEDED_FILE.SAM -t 8 FILTERED.FA GENOME’ and ‘delve align -u 1 -o ALIGNED.BAM -t 8 SEEDED_FILE.SAM GENOME’*

SEEDED_FILE.SAM is the output seeded file from FILTERED.FA, ALIGNED.BAM is the final alignment and GENOME is the genome assembly used to map the reads to.

The first command is used to seed the alignment, with a seed length of 12 bases (-l option), step size 8 (-s) and with max 8 threads (-t). The second command is used to build the HMM model and assign the scores to the aligned reads. Option -u ensures that only one alignment is reported (with the highest score).

Expression quantification was performed as described previously^16^. Simply, frequencies of the observed transcription start sites (TSSs) at a single base resolution were extracted from the alignments by using a combination of samtools^29^ and bedtools^30^, such that all mapped CAGE reads aligning at the same 5′ position accounted for the expression of the TSS at that position. We retained only aligned reads with 99% accuracy (mapping quality q>20) and with sequence identity above 85%. In practice, each alignment, for both dog and rat, was processed as follows:

*‘samtools view -q 20 -F 768 -u ALIGNED.BAM|bamToBed -i stdin| awk 'BEGIN{OFS="\t"}{if($6=="+"){print $1,$2,$5}}'| sort -k1,1 -k2,2n| groupby -i stdin -g 1,2 -c 3 -o count| awk 'BEGIN{OFS="\t"}{print $1,$2,$2+1, $1":"$2".."$2+1",+",$3,"+"}'*

The commands ensure that the reads are filtered for any other bad alignments (using the flag value 768) that may still pass the mapping quality threshold, then converted to a bed file. TSS-level expression is then obtained by grouping, and counting, the reads with the same start position (done for plus and minus strand orientation separately, here shown only for ‘+’ strand). The total number of uniquely mapped reads for each sample is given in Supplementary Table 1. Aligned reads (BAM format) and 1-base resolution frequencies of transcription initiation (BED format) are publicly available via DDBJ (see Data Records, Data Citations 1 and 2).

### Identification of promoter regions

CAGE peaks were defined by using a Decomposition Peak Identification (DPI) method as described previously^16^. Briefly, regions of continuous, composite signal were identified genome-wide, were subsequently decomposed across samples in order to discriminate the signal coming from distinct samples, and finally peaks were called based on distance and a minimum read counts metrics. Peaks were obtained by running the software with default setting, taking as input the BED files of 5′-end frequencies produced as described above. Two sets of peaks were generated: a ‘permissive’ set of promoters with minimum 3 read counts in a single position in at least one sample, and a ‘robust’ set with minimum 1 tpm and 10 read counts in a single position in at least one sample. This ensured that the sets of peaks for dog and rat were comparable to those generated for human and mouse^16^. Since HeliScopeCAGE is an amplification-free protocol based on single-molecule sequencing, individual reads represent independent evidence of capped 5′-ends. In defining the peak sets, read counts corresponding to the number of observations were primarily employed as a threshold, and the expression level (1 tpm threshold) was added to ensure the robust set included only peaks with a certain minimum level of expression.

The peaks were annotated based on Ensembl transcripts, Augustus, GeneScan, GeneID, RefSeq and EST gene models obtained from UCSC genome browser^22^ (download date 06/2016). As an annotation rule we followed what was done previously^16^, that is a CAGE peak overlapping a 1 kb region centred on the gene’s 5′ end on the same strand orientation is associated to that gene.

### Projections of human promoters to dog and rat

The conversion of the promoters’ genomic coordinates from human to dog and rat was performed using liftOver tool (see Code availability). Default parameters with pairwise alignment chain files ‘hg19ToCanFam3.over.chain.gz’ and ‘hg19ToRn6.over.chain.gz’ were used to convert human coordinates into rat and dog (downloaded from the UCSC site http://hgdownload.cse.ucsc.edu/goldenPath/hg19/liftOver/). In total, 129,287 (64%) and 111,218 (55%) human CAGE promoters could be projected to dog and rat, respectively. In order to make sure that we considered likely conserved promoters only, we required that a projected promoter be within 50 bp both upstream and downstream of the promoter in the destination genome, following a similar rationale as applied in Young *et al.*^31^. Past works on promoter architecture and evolution across species showed that conservation decreases beyond a 70 nt distance^32^. Moreover, since the set of human CAGE peaks was obtained from profiling thousands of samples, we filtered the set to only use those likely conserved peaks that were expressed in the corresponding cell types to rat and dog. A summary of the number of projected promoters is given in Table 1.

### RNA-seq data processing

Publicly available RNA-seq data were downloaded for dog (Data Citations 3 to 5) and rat (Data Citation 6). The rat (Fisher 344 strain) data sets comprise tissues sampled from 11 different organs (muscle, spleen, brain, uterus, testis, kidney, heart, thymus, lung, liver, and adrenal gland), of both sexes and at varying development stages (2 to 100 weeks old). We arbitrarily chose three female and three male samples for each tissue origin, with the exceptions of uterus and testis. RNA-seq samples for dog represented heart (normal and diseased), pituitary, adrenal cortex, and lymph node tissues (normal and B-cell lymphoma). Ages of the dog samples used in the heart disease study varied from 3 to 17 years, and pituitary and adrenal samples were collected from adult dogs; we couldn’t find age information for the lymphoma study samples. A summary of the data sets used is given in Supplementary Table 2 for rat and Supplementary Table 3 for dog.

Data sets were reprocessed using HISAT2, an improved version of HISAT tool^33^, to align them to the canFam3 and rn6 reference genomes.

### Additional data processing

Genomic distribution of the CAGE peaks and screening for TATA-box motifs was performed via HOMER^34^ (v4.8.1) using the ‘annotatePeaks.pl’ function with standard parameters, except for the TATA motif annotation where a region 500 bp upstream and 200 bp downstream around the peak was scanned. The HOMER default reference gene set for genomic distribution annotations is RefSeq, and the default promoter region window is from 1,000 bp upstream to 500 bp downstream of a gene TSS:

*‘annotatePeaks.pl PEAK_FILE.BED GENOME -size -500,200 -m data/knownTFs/motifs/tata.motif -multi -CpG -noann>tata_annotation.txt’*

*‘annotatePeaks.pl PEAK_FILE.BED GENOME -size given -go DEST_DIR -genomeOntology DEST_DIR -annStats stats_record.txt>annotation.txt’*

Student’s *t*-test was performed on the two sets of CpG- and TATA-associated promoters using the R language function t.test() with default parameters. Clustering of samples was performed using R bioconductor edgeR package^35^. Specifically, normalization of expression values was calculated with the function ‘calcNormFactors()’ and the plot was visualized with the function ‘plotMDS()’. Figures were all generated using R, version 3.1.3.

### Genomic views

All our data is visualized via our original genome browser ZENBU^36^. Pre-configured views for rat and dog sets can be accessed at these URLs:

http://fantom.gsc.riken.jp/zenbu/gLyphs/#config=3eolUxzyN1nm167YHayYLC (rat)

http://fantom.gsc.riken.jp/zenbu/gLyphs/#config=ddICFtA-H1upfbn6qjjFID (dog)

### Code availability

Mapping was performed by our own algorithm delve, version 0.95, described in Djebali *et al.*^37^ specifically developed for handling data derived from HeliScope single molecule sequencing. The software is available at this URL (fantom.gsc.riken.jp/5/suppl/delve/).

The DPI peak calling was performed using our own software, detailed in Forrest *et al.*^16^. It can be freely downloaded at this URL (https://github.com/hkawaji/dpi1/).

The liftOver tool was downloaded from the UCSC software repository^38^ (http://hgdownload.soe.ucsc.edu/admin/exe/).

The samtools (v1.3) and bedtools (v2.23.0) packages^29,30^ were downloaded from (https://sourceforge.net/projects/samtools/ and https://github.com/arq5x/bedtools2).

The HISAT package^33^ (v2.0.5) was downloaded from (https://github.com/infphilo/hisat).

The HOMER^34^ motif discovery tool (v4.9) can be found at the following URL (http://homer.salk.edu/homer/download.html).

## Data Records

The data resulting from sequencing (fastq format), mapping (BAM format) and TSS profiling (BED format) can be found in DDBJ data repository (Data Citations 1 and 2). The same datasets, together with the identified CAGE peaks sets and association to gene models can also be found within the FANTOM data repository (http://fantom.gsc.riken.jp/5/datafiles/latest). The folder ‘basic’ contains the primary mapping data (BAM format) and TSSs (BED format). The folder ‘extra’ contains the subfolder ‘CAGE_peaks’ for the coordinates of the identified peaks (BED format), peak-based expression tables for raw and normalized counts (tabular text format) and associations to gene models (tabular text format). A detailed description of the samples used in this study, such as cell type, strain, species, tissue of origin, is also given in Supplementary Table 1.

## Technical Validation

### Primary cell data show high reproducibility across replicates

**Figure 2:**
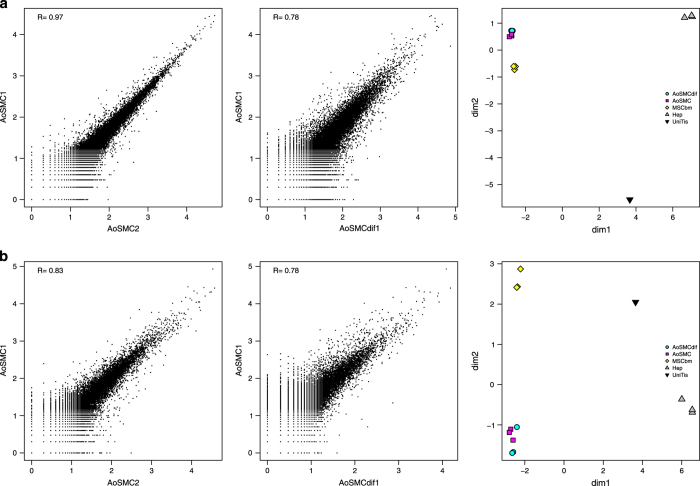
Reproducibility of replicates. Scatter plots and correlation values of normalized expression values between AoSMC samples (replicate 1 versus replicate 2 on the left and replicate 1 differentiated versus non differentiated in the center), and MDS plots highlighting the separation across cell types are shown for rat (**a**) and dog (**b**).

Biological replicates of each cell type were tested in order to assess the reproducibility of the experiments. Results show agreement within all three replicates for all cell types (Table 2), as seen, for instance, for two of the rat aortic smooth muscle (AoSM) cells replicates where Spearman correlation is 0.97 (Fig. 2a). The same level of reproducibility is evident also in the dog primary cell replicates (Fig. 2b), although we obtained less amount of RNA for one of the dog AoSM cell replicate. That forced us to adopt a variation of the standard library preparation protocol (see Methods), which resulted in a lower total read count and consequently in a slightly lower correlation (0.83, Spearman).

The correlation between the differentiated and genuine AoSM cells is still relatively high, whereas it decreases when AoSM cells are compared to phenotypically distinct cell types such as hepatocytes, which means that the CAGE expression alone is already enough to distinguish between those two states. Clustering analysis via multi-dimensional scaling (see Methods) shows that samples group based on cell type (Fig. 2).

### Characterization of the dog and rat CAGE promoters

**Figure 3:**
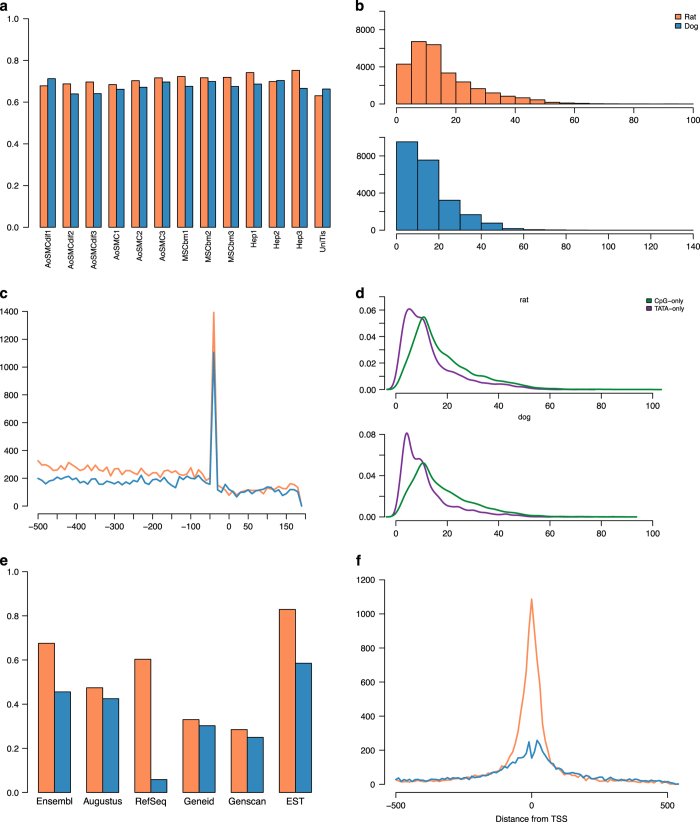
Characterization of CAGE peaks in dog and rat. (**a**) Percentage of mapped reads at promoters identified by DPI for each sample. Labels description: AoSMC=aortic smooth muscle cell; AoSMCdiff=differentiated aortic smooth muscle cell; MESbm=mesenchymal stem cell from bone marrow; Hep=hepatocyte; UniTis=Universal tissue; (**b**) histograms of CAGE peaks lengths; (**c**) enrichment of TATA motifs near CAGE peaks; (**d**) graphs showing TATA-rich versus CpG-rich peaks. TATA-only bound CAGE peaks tend to be sharp whereas CpG-only peaks are generally broader; (**e**) percentage of genes that can be associated to a CAGE peak for each of the inspected known models; (**f**) distribution of the distances of CAGE peaks from their closest gene TSS. Colours: orange denotes rat and blue dog, except for (**d**), where colour-code is specified in the legend.

All samples were sequenced on HeliScope single molecule sequencer^27^. While the number of mapped reads across samples is comparable in general, many factors can affect it, like RNA amount, RNA quality, protocol efficiency or sequencing errors. The total number of mapped reads for the rat samples varied between 1 M (mesenchymal stem cell donor 2 and 3) and 7 M (AoSM cell), while the universal RNA tissue sample topped 10 M uniquely mapped reads. Uniquely mapped reads for dog samples were, as stated above, generally lower than for rat but with some exceptions, varying between 500,000 of mesenchymal stem cells and 10 M of hepatocytes (Supplementary Table 1). We observed a consistent lower number of uniquely mapped reads for the mesenchymal stem cells in both species, compared to the other cell types. At any rate, the percentage of mapped reads at putative TSSs is more or less constant, around 70% (Fig. 3a), suggesting that the majority of the CAGE signal is concentrated around specific genomic locations (i.e., CAGE peaks) identifying genuine transcription initiation rather than being scattered everywhere.

We calculated the CAGE based expression levels in each sample separately, and then aggregated the signal into CAGE peaks representing transcription initiation events, or promoters (see Methods). The total number of CAGE peaks is comparable across species: we obtained 28,497 and 23,147 ‘robust’, and 92,031 and 85,324 ‘permissive’ promoters for rat and dog, respectively. Unless explicitly stated, all the analyses presented in this work are based on the robust set of promoters only.

One of the main points of the DPI method in identifying peaks is the ability to decompose big regions of CAGE signal into smaller, non overlapping segments with expression. In order to verify such ability, we calculated the distribution of CAGE peaks sizes and confirmed that they were a) short and b) were consistent between species. Distributions of peak sizes show that they tend to be less than 150 bp long, with the majority of them being 10 to 30 bp (Fig. 3b). By comparison, human and mouse promoters can be longer, up to 300 and 200 bp, respectively^16^, but the majority of them are around 20–25 nucleotides long, resembling the distributions in rat and dog datasets.

Finally, we evaluated the ratio of CpG- and TATA-rich regions. Promoters are generally classified as either sharp, TATA-rich, shorter regions that harbour most of the expression within a few nucleotides, or broad, CG-rich ones where the expression is distributed on a larger region with sub-peaks of sharp expression^32,39^. Moreover, TATA-rich promoters tend to be associated to housekeeping genes^16,32^. CpG islands are one of the most prominent indications of promoters^32^, and indeed we found 57% (16,327) and 60% (13,863) of rat and dog CAGE peaks, respectively, overlapping a CpG. Since there is no publicly available dataset for TATA-rich regions in dog and rat, we used HOMER motif finding tool^34^ to check for enrichment of TBP (TATA binding protein) sites around our promoters. An appreciable number of CAGE peaks harboured TATA-box motifs in the region 500 bp upstream and 200 bp downstream of the peak region (32.7% (9,338) and 29% (6,747) in rat and dog respectively), with a clear preference for the region 30–35 bp upstream of a TSS (Fig. 3c). We next subdivided the CAGE peaks into three categories, TATA-only (3,775 dog and 4,763 rat), CpG-only (10,891 and 11,752 dog and rat respectively) and both, and compared their sizes. Although it is not as evident as in human promoters, we could observe that TATA-only promoters tend to be shorter than the CpG-only ones (Student’s *t*-test, *P*-value<2.2e-16 in both species) (Fig. 3d). These results are in line with what is known for other model organisms^13,16^.

### CAGE can contribute to refine the promoter landscape of poorly characterized genomes

In order to have a rough estimate of what genomic features the CAGE promoters are closest to, we took the dog and rat peaks and annotated them by using HOMER’s annotation tool (see Methods). We found that 58% (16,625) of rat CAGE peaks are located near TSSs of known genes (Table 3). In the case of dog CAGE peaks, the picture that emerged was different, with only 1,394 peaks annotated as promoters of known RefSeq genes, while the majority of them were annotated as CpG-island (Table 3), highlighting the fragmentary coverage of the dog genome. In fact, the total number of RefSeq genes for dog is as low as about 2,300. As we would like to employ CAGE as an accessory means to improve resolution, coverage and accuracy of the existing gene models, we first downloaded all available gene models, either manually curated or predicted, from UCSC genome browser^22^ for both dog and rat, and associated them to the CAGE peaks (see Methods) to see how many we could recover.

For the rat CAGE peaks, we could link 19,254 of them (68%) to the TSS of a known Ensembl transcript and 17,188 (60%) to that of a known RefSeq gene (Fig. 3e). Distributions of the distances between all CAGE-defined TSSs and the genes’ TSSs show that CAGE technology captures loci of genuine transcription initiation (Fig. 3f). For 2,289 (8%) CAGE peaks we found no association to any of the gene references. Of those, however, 1,761 (77%) were overlapping with one or more of CpG islands (harbouring the potential for novel TSSs), repeats, gene bodies (i.e., introns or exons more than 500 bp downstream of the TSS). The remaining 520 un-annotated peaks are either intergenic, potential bidirectional enhancers^11^, or are located more than 500 bp upstream of a known gene TSS.

In the case of the dog dataset we could associate 10,542 (46%) of the CAGE peaks to Ensembl transcripts, and only 1,350 (6%) to known RefSeq genes (Fig. 3e). The large difference is likely due to gene coverage, as only ~2,200 genes are annotated by RefSeq, which adopts strict experimental validation criteria^40^, against the ~39,000 transcripts (corresponding to about 29,000 genes) reported in Ensembl. On the other hand, the proportion of Ensembl transcripts covered by a CAGE peak in dog is as low as 21% (Table 4), not much lower than what observed in rat (27%). This, and the fact that CAGE peaks tend to align at the TSS of known genes (Fig. 3f) together suggest likely incorrect or incomplete gene annotations. The number of un-annotated peaks for the dog data set was 4,550 (19%), with 873 of them found far from other known features. The peaks-to-genes associations are summarised in Table 4, while the total numbers of CAGE peaks associated to zero or more gene models are listed in Table 5.

To check whether we could increase the gene annotation rate, we converted the coordinates all human robust CAGE promoters identified in a previous study^16^ to both dog and rat genomes via the liftOver tool (see Methods). These human projections would thus serve to confirm the already annotated peaks and reduce the number of the un-annotated ones, in a similar manner to a guilt-by-association exercise. We chose the human CAGE promoters set primarily because it is the most accurate, comprehensive and characterized, but also because a lift-over of full transcripts to other species is error-prone due to length, splicing, or directionality of genes. For all peaks, we adopted a distance rule requiring the human projected peak be within 50 bp of a dog or rat peak in order to consider the alignments reliable (see Methods). Overall, 79% of dog and 67% of rat CAGE peaks were aligned with a projected human peak expressed in the same cell types (Table 1). Regarding the un-annotated peaks, we required in addition that their aligned human peak be annotated and within 10 kb of a dog or rat orthologous gene to be eligible for annotation. We used Ensembl genes for this survey. Following this principle, we isolated 205 and 801 unique rat and dog peaks respectively, and named them Rescued CAGE Peaks (RCP) (Data Citation 7).

As an additional support, we also used publicly available RNA-seq data for dog and rat (Data Citation 3 to 6), as close to the cell types we profiled as possible, and used them to check whether the RCP are located at the 5′-end of the RNA-seq model as well. Even though it is not a general rule, we’ve noticed several cases where if the RCP are near the dominant human peak (most expressed, noted as ‘p1@gene’) they exhibit higher expression, the RNA-seq model supporting the gene is better defined and chances of them representing a better TSS for the gene are higher.

**Figure 4:**
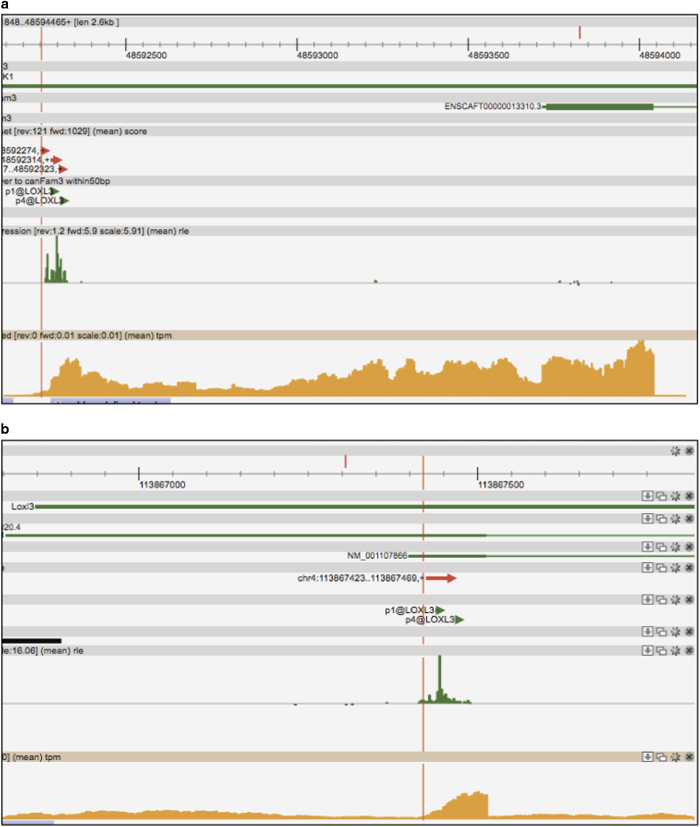
Zenbu examples of Rescue CAGE Peaks. Screen shots of (**a**) *LOXL3* gene in dog with RCPs supported by RNA-seq and human lift-over promoters, and (**b**) *Loxl3* gene in rat annotated with CAGE peaks, also supported by RNA-seq and human promoters.

One example is *LOXL3* gene, which was not associated to any CAGE peak in dog because the TSS of its corresponding Ensembl transcript (ENSCAFT00000013310.3) is more than 1 kb away; however, there are two lifted-over human *LOXL3* peaks within 50 bases of the un-annotated dog CAGE peaks (Fig. 4a). RNA-seq data shows that the 5′ end of *LOXL3* gene is indeed further upstream than the current defined model, judging by the drop of signal at the start of the CAGE peak. As a comparison, we checked the rat genome, where *Loxl3* gene is associated to a CAGE peak, instead, and exhibits a similar RNA-seq profile defining the 5′ end together with the same human *LOXL3* peaks (Fig. 4b).

We tried to find out whether those RCP or their associated genes had some features in common. We observed that all RCP in both dog and rat were relatively short, with a median size of 11 bp and tend to be expressed at low levels (Data Citation 7). We next looked at the RCP nearby genes, noticing that, for the vast majority, they are very big, with a median length of 80 kb in rat and 65 kb in dog (Data Citation 7). Since Ensembl genes are predicted, many of them may represent mere open reading frames (ORFs), and in that case the reported TSS would not represent the real one, but just the beginning of the ORF; this scenario could explain why several peaks could not be associated to genes in the first place. We checked this by calculating the distance between the genes’ transcription and coding sequence start, which was zero for 48% (301/622) and 34% (68/201) of dog and rat RCP associated genes (Data Citation 7). In comparison, of the genes associated to a CAGE peak only 12.5% of them in rat are ORFs, while for dog, where the number of curated genes (i.e., RefSeq) is overall lower, the proportion is 52%. Alternatively, the observation that most genes were associated to multiple RCP found within their body could signify the existence of previously unknown isoforms^13,16,32^ (i.e., *Ncor2* gene in rat, http://fantom.gsc.riken.jp/zenbu/gLyphs/#config=3eolUxzyN1nm167YHayYLC;loc=rn6::chr12:36831460..37074148+). Relative to the type of the RCP associated genes, we found that 25% (36/147) of those in rat and 32% (155/478) in dog are known TFs in human; 8 TF genes (*AHR, AHRR, HOXB6, JARID2, KDM2A, NCOR2, TSC22D2, ZC3H4*) are listed in both RCP sets.

This simple survey showed that, although a systematic validation of all RCP should be pursued, CAGE profiling can be considered as a useful tool to refine existing gene models, even with a limited number of cell types at disposal.

## Usage Notes

The FANTOM web portal (fantom.gsc.riken.jp) provides a starting point for data download and exploration. Genomic coordinates and frequencies of TSSs at a single base-pair resolution can be found in fantom.gsc.riken.jp/5/datafiles/latest/basic/, whereas the CAGE peaks, their expression across samples and their gene annotation can be found in fantom.gsc.riken.jp/5/datafiles/latest/extra/. Transcription initiation signals can also be used to identify transcribed enhancers^11^; however, it has to be noted that the signal from enhancers are quite weak in general. Given the quite limited number of samples, it was not possible to identify enhancer profiles in the two species dog and rat.

Visual inspection of CAGE peaks positions and expression patterns, in relation to gene models and other genomic elements is possible via ZENBU genome browser (fantom.gsc.riken.jp/zenbu/), offering a quick and interactive overview of a genome’s complex structure, as well as via a FANTOM5 UCSC track hub (https://genome.ucsc.edu/cgi-bin/hgHubConnect?hubUrl=http%3A%2F%2Ffantom.gsc.riken.jp%2F5%2Fdatahub%2Fhub.txt). Promoter-level expression based on the CAGE peaks can be easily obtained by using the Table Extraction Tool (TET, fantom.gsc.riken.jp/5/tet/). Finally, CAGE peak centric page and sample centric ones are accessible in SSTAR semantic browser (fantom.gsc.riken/5/sstar/).

All data available through the web portal are public and thus free to use.

## Additional Information

**How to cite this article:** Lizio, M. *et al.* Monitoring transcription initiation activities in rat and dog. *Sci. Data* 4:170173 doi: 10.1038/sdata.2017.173 (2017).

**Publisher’s note:** Springer Nature remains neutral with regard to jurisdictional claims in published maps and institutional affiliations.

## Supplementary Material



Supplementary Table 1

Supplementary Table 2

Supplementary Table 3

## Figures and Tables

**Table 1:** Human (hg19) CAGE peaks liftOver to dog and rats.

	**Robust expressed**	**Robust within 50 bp**	**Within 50 bp and expressed in matching samples**	**Total hg19 CAGE peaks**
hg19 -> canFam3	129,287	19,302	18,374	201,802
hg19 -> rn6	111,218	20,742	19,096	201,802
Reported are the numbers of: expressed lifted over human peaks, peaks within 50 bp of a dog/rat peak, peaks expressed in matching dog/rat samples. Total of human peaks are also reported, for reference.				

**Table 2:** Matrix of correlation values for all pairwise comparisons of dog and rat samples.

	**AoSMCdiff1**	**AoSMCdiff2**	**AoSMCdiff3**	**AoSMC1**	**AoSMC2**	**AoSMC3**	**MSCbm1**	**MSCbm2**	**MSCbm3**	**Hep1**	**Hep2**	**Hep3**	**UniTis**	**Dog**
AoSMCdiff1		0.89	0.91	0.78	0.87	0.84	0.58	0.58	0.65	0.06	0.06	0.08	0.2	AoSMCdiff1
AoSMCdiff2	0.91		0.98	0.68	0.9	0.88	0.47	0.45	0.53	0.05	0.04	0.06	0.16	AoSMCdiff2
AoSMCdiff3	0.74	0.9		0.72	0.9	0.87	0.53	0.52	0.59	0.05	0.04	0.06	0.16	AoSMCdiff3
AoSMC1	0.78	0.73	0.76		0.83	0.72	0.83	0.85	0.84	0.1	0.08	0.11	0.24	AoSMC1
AoSMC2	0.78	0.71	0.73	0.97		0.94	0.64	0.64	0.72	0.07	0.06	0.08	0.21	AoSMC2
AoSMC3	0.75	0.72	0.8	0.92	0.94		0.48	0.48	0.59	0.04	0.04	0.05	0.17	AoSMC3
MSCbm1	0.6	0.49	0.49	0.7	0.77	0.75		0.98	0.95	0.1	0.08	0.11	0.2	MSCbm1
MSCbm2	0.6	0.49	0.52	0.74	0.81	0.81	0.95		0.97	0.1	0.08	0.11	0.2	MSCbm2
MSCbm3	0.48	0.4	0.42	0.66	0.72	0.65	0.77	0.81		0.09	0.07	0.1	0.2	MSCbm3
Hep1	0.03	0.06	0.07	0.04	0.03	0.04	0.01	0.01	0		0.98	0.96	0.72	Hep1
Hep2	0.04	0.07	0.08	0.05	0.03	0.05	0.01	0.01	0.01	0.99		0.99	0.76	Hep2
Hep3	0.03	0.04	0.05	0.03	0.02	0.03	0.01	0.01	0	1	0.99		0.77	Hep3
UniTis	0.1	0.14	0.16	0.13	0.11	0.13	0.06	0.06	0.05	0.92	0.92	0.92		UniTis
Rat	AoSMCdiff1	AoSMCdiff2	AoSMCdiff3	AoSMC1	AoSMC2	AoSMC3	MSCbm1	MSCbm2	MSCbm3	Hep1	Hep2	Hep3	UniTis	
The top right side of the table shows pairwise Spearman correlation values for the dog samples, while the bottom left side shows corresponding values for the rat samples. Column and row labels abbreviation: AoSMCdiff—aortic smooth muscle cells differentiated; AoSMC—aortic smooth muscle cells; MSCbm—Mesenchymal stem cells bone marrow derived; Hep—hepatocytes; UniTis—universal RNA tissue. Numbers 1,2,3 indicate biological replicates.														

**Table 3:** Number of dog and rat CAGE peaks overlapping known genomic features, as annotated by HOMER tool.

	**Number of CAGE peaks**	
**Annotation based on HOMER**	**Rat**	**Dog**
3UTR	474	111
ncRNA	18	0
TTS	403	209
LINE	82	286
SINE	114	122
tRNA	1	0
DNA	17	65
Exon	1,937	513
Intron	1,561	163
Intergenic	3,013	8,001
Promoter	16,625	1,394
5UTR	1,054	57
scRNA	4	0
CpG-Island	2,770	11,321
Low_complexity	28	427
LTR	169	114
Simple_repeat	122	213
snRNA	34	3
Unknown	2	0
Satellite	2	1
rRNA	63	146
srpRNA	0	1
RefSeq gene is the reference set used by the tool. 3UTR=3-prime untranslated region; ncRNA=non-coding RNA; TTS=transcription termination site; LINE=long interspersed nuclear element; SINE=short interspersed nuclear element; tRNA=transfer RNA; 5UTR=5-prime untranslated region; scRNA=small cytoplasmic RNA; LTR=long terminal repeats; snRNA=small nuclear RNA; rRNA=ribosomal RNA; srpRNA=signal recognition particle RNA.		

**Table 4:** Totals and ratios of peaks-genes associations.

	**Totals**		**CAGE peaks annotated**		**CAGE peaks annotated (%)**		**Genes covered by CAGE**		**Genes covered by CAGE (%)**	
	**Rat**	**Dog**	**Rat**	**Dog**	**Rat**	**Dog**	**Rat**	**Dog**	**Rat**	**Dog**
Ensembl_transcript	39,595	29,881	19,254	10,542	68%	46%	1,0688	6,194	27%	21%
Augustus_gene	29,380	29,165	1,3513	9,829	47%	42%	7,328	5,755	25%	20%
RefSeq_transcript	18,978	2,274	17,188	1,350	60%	6%	9,246	779	49%	34%
Geneid_gene	41,652	32,342	9,399	6,997	33%	30%	5,339	4,155	13%	13%
Genscan_gene	49,319	42,671	8,115	5,779	28%	25%	4,618	3,525	9%	8%
EST_gene	1,270,134	401,654	23,621	13,549	83%	59%	13,718	7,932	1%	2%
All robust CAGE peaks	28,497	23,147	26,208	18,597	92%	80%	28,497	23,147	100%	100%
Listing of the totals and percentages for the gene models used in this study together with all robust peaks identified in this study in dog and rat.										

**Table 5:** Breakdown of numbers of CAGE peaks overlapping zero or more gene models.

	**CAGE peaks per gene annotation sets**						
	**0**	**1**	**2**	**3**	**4**	**5**	**6**
Rat	2,289	5,045	2,646	5,000	5,617	4,115	3,785
Dog	4,550	5,984	3,943	3,202	3,004	2,230	234
The gene model sets refer to those listed in Table 4 (Ensembl, RefSeq, Augustus, Geneid, Genscan, EST).							
